# Help on Demand, a Self-Directed Mobile App Intervention for Gambling Problems: Development and Usability Study

**DOI:** 10.2196/83430

**Published:** 2026-03-24

**Authors:** Brad W Brazeau, John A Cunningham, David C Hodgins

**Affiliations:** 1 Department of Psychology, University of Calgary, 2500 University Dr NW, Calgary, AB, T2N1N4, Canada, 1 403-210-9500; 2 National Addiction Centre, Institute of Psychiatry, Psychology, and Neuroscience, King's College London, London, United Kingdom; 3 Institute for Mental Health Policy Research, Centre for Addiction and Mental Health, Toronto, ON, Canada; 4 Department of Psychiatry, University of Toronto, Toronto, ON, Canada

**Keywords:** gambling, addictive behavior, ecological momentary intervention, just-in-time adaptive intervention, mobile app, telemedicine, self-help, self-directed treatment

## Abstract

**Background:**

Compared with other mental health problems, self-directed interventions for gambling problems lack in quantity, accessibility, and in some cases, evidence base. Moreover, engagement with these interventions remains modest. Mobile apps may be a viable format to deliver self-directed interventions that enhance user engagement.

**Objective:**

The aims of this study were to develop a self-directed mobile app intervention for gambling problems and to conduct initial feasibility and acceptability testing with a small sample of Canadian adults with past or present gambling problems (n=30).

**Methods:**

Participants were invited via email from a list of people who had previously volunteered in similar research in our laboratory. Theory and content of the mobile app intervention were primarily based on a self-directed workbook that has been evaluated in paperback and static web-based formats. The current app prototype included daily gambling diaries, recommended activities based on diary responses, and psychoeducation. It was available for 2 weeks, after which users provided feedback via surveys (n=30) and a virtual focus group (n=8). Quantitative and qualitative feedback, as well as app usage data, were analyzed to provide descriptive statistics and summaries.

**Results:**

Regarding feasibility, median completion time for activities ranged from 48 (IQR 35-90) to 137 (IQR 93-328) seconds. Daily diary completion rate was 51%. One-third of activities were accessed via prompt, and two-thirds on demand. Many participants repeated at least 1 activity, and all activities were repeated by at least 1 participant. Results also indicated favorable user reviews, particularly regarding the app’s credibility, ease of use, and potential impact. The feedback on some app features was highly variable, such as the perceived utility of daily diaries. Specific recommendations for improvement were provided, such as the inclusion of information on concurrent substance use and more interactive psychoeducation.

**Conclusions:**

Overall, the app met or exceeded heuristic thresholds for feasibility and acceptability testing. These results will inform improvements and subsequent effectiveness testing. The variability in user feedback underscores the demand for further personalization.

## Introduction

### Background

Those who seek self-directed treatment for many mental health problems are spoiled for choice. In fact, there is so much choice for some disorders that treatment selection itself can be overwhelming [1]. Paper-and-pencil workbooks, web programs, and mobile apps permeate the self-help space, particularly for mood and anxiety disorders [2-4]. Generic mental health apps, including Headspace (Headspace Inc) and Calm (Calm.com, Inc), are also highly popular [5,6]. People with gambling problems are not nearly as well-supported in their specific needs, some of which are predominant in gambling, such as financial concerns. This gap is unfortunate, given that there is a high demand for self-directed gambling treatment. Most gamblers who attempt to change do so independently [7], and many prefer to use self-directed interventions for the privacy, ease of access, and ability to control their pace and level of engagement [8-11]. The current paper describes and contextualizes the development of a self-directed, tailored mobile app for gambling problems specifically designed to enhance user engagement.

Evidence-based self-directed treatment for gambling problems first took hold in the 1990s and 2000s, mainly in the form of cognitive behavioral bibliotherapy [12]. In the 2010s, much of the same content was delivered via the web to expand accessibility and cost-effectiveness [13]. Multiple theories and treatment approaches were implemented in these resources, many of which were offshoots of cognitive behavioral therapy (CBT) or third-wave modalities. Newer technologies, like mobile apps, virtual reality, and artificial intelligence (AI), have become a central focus of self-directed interventions for gambling problems in the 2020s [14]. These emerging modes of delivery are especially promising because, in addition to offering all the benefits of 2010s-style web-based interventions, they increasingly permit tailored, responsive, and interactive content rather than static, generalized, or read-only resources. As it stands, however, very few evidence-based options are publicly available to treatment-seeking gamblers.

In a review, Merkouris [15] identified 6 evidence-based mobile apps for gambling problems. Most were based on preexisting paperback or web-based workbooks (eg, study by Dowling et al [16]). However, only 1 French-language app was publicly available. The most thoroughly researched app, GamblingLess: Curb Your Urge (Deakin University), specifically targeted transient gambling urges with an array of cognitive, behavioral, and mindfulness-based strategies [17,18]. Preliminary results indicated good acceptability and effectiveness, albeit low uptake and compliance. The same research group has published protocol papers for the development of similar mobile apps that vary in their intended audience [19-21]. Other mobile apps for gambling problems are publicly available, but most are solely abstinence-based and do not rely on a recognizable treatment model [22]. For example, most available apps consist only of an abstinence time tracker, while some also include reminder notifications and motivational quotes. Some newer apps contain gambling website blocking software, gambling-related information, and directories of external support services, but very few were developed in conjunction with people with lived experience [23]. Taken together, there are not many viable mobile apps that target gambling problems, but preliminary research on a select few is encouraging.

Perhaps the greatest challenge in offering self-directed interventions for mental disorders, including gambling problems, has been low user engagement [24]. Engagement in this context is often measured in terms of objective and subjective indicators of treatment use, such as the number of activities completed or ratings of subjective appeal [11,25]. Engagement is not simply adherence to an intervention, although adherence is often considered one piece of engagement [26,27]. Rather, engagement likely comprises a combination of behavioral, cognitive, and affective factors, including enjoyment, motivation, and perceived relevance [27,28]. Perski and colleagues [11] proposed that subjective and objective engagement is moderated by the content and delivery of the intervention, the real-world context of its use, and the behavior it targets. Treatment outcomes are, in turn, moderated by user engagement. The recent development of tailored and adaptive interventions has been, in large part, a response to the problem of low engagement by improving intervention content and delivery.

The problem of low user engagement has been underscored in the gambling field, as intervention usage data has revealed just how modest user engagement tends to be. In our recent trial of a static self-directed web program for problem gambling [29], we found that 41% (128/313) of participants did not complete any modules, but those who completed more modules experienced greater reductions in gambling problems. Similar findings were documented in our own past work [30] and that of others [15]. This general observation is somewhat surprising given how positively users rate such interventions, and how self-directed change directly addresses barriers to traditional face-to-face treatment, such as stigma and lack of privacy [9,10].

### Study Objectives

The primary aim of this study was to contribute to the growing literature on evidence-based mobile apps for gambling problems by developing a personalized self-directed mobile app intervention grounded in content from our previously developed static workbook. The intervention was designed to facilitate greater engagement relative to static self-directed interventions by making the content more appealing and the delivery more responsive to individual needs. We aimed to conduct initial feasibility and usability testing with a small sample of Canadian adults with past or present gambling problems and solicit their feedback to inform subsequent improvements and testing. Given the formative nature of this research, effectiveness and longer-term engagement were not assessed at this stage.

## Methods

### Ethical Considerations

This study received ethical approval from the Conjoint Faculties Research Ethics Board at the University of Calgary (REB23-0794). This approval authorized participant recruitment, informed consent, collection and storage of app usage and survey data, and participation in a focus group on Microsoft Teams. Participants were remunerated with up to CAD $40 (US $29.46) in electronic gift cards for their participation. Deidentified data from Qualtrics, *MetricWire* (MetricWire Inc)*,* and Microsoft Teams were stored separately from personally identifying information in an encrypted file.

### Participants

Canadian adults with past or present gambling problems were recruited over 3 months via email contact from researchers. These email invitations were sent to 100 participants from a previous gambling self-help trial [29] who had volunteered to be contacted for future research. We received 71 responses to an eligibility survey on Qualtrics, of which 33 were eligible, consented, and enrolled. Participants were eligible if they resided in Canada, were aged 18 years or older, and scored 5 or greater [31] on the Problem Gambling Severity Index (PGSI) [32]. Those with past problems were asked to respond to the PGSI based on their gambling at the peak of their problem instead of the typical past-year time frame. This approach was used to ensure participants without current gambling problems previously had a problem severity consistent with the app’s likely audience, that is, people with moderate to severe gambling problems [33].

Most of those excluded from this study were excluded because they did not complete the eligibility survey in full or did not respond to the initial email contact after they were found to be eligible. Furthermore, 3 participants were subsequently dropped because they did not create an account with the mobile app and did not respond to follow-up emails.

### Procedure

Enrolled participants were emailed a link and brief instructions to download and register with the third-party app, labeled *Catalyst by MetricWire,* in the Apple App Store and Google Play Store. Users interacted with our intervention via the *Catalyst* mobile app on which the intervention was mounted. We selected this platform to host our intervention because it was used successfully by our colleagues to develop a similar gambling intervention in Australia [16,18].

Participants were not given specific instructions for engaging with the app content but were informed that it was still under development, and the researchers would be interested in their feedback. Participants had access to the app for 2 weeks, after which they completed a brief feedback survey on Qualtrics and were compensated with an electronic gift card valued at CAD $20 (US $14.73). Then, 8 participants were randomly selected via a random number generator and invited to participate in a focus group facilitated remotely by author BWB and 2 research assistants on Microsoft Teams. Focus group discussions used a semistructured interview guide (Multimedia Appendix 1) to elaborate on user experiences with the app as well as their quantitative feedback. Because of ethical constraints, participants’ survey responses and focus group contributions were not linked to one another. Participants who provided written or oral feedback were compensated with an additional electronic gift card valued at CAD $20 (US $14.73).

### Intervention

#### Theoretical Framework

The intervention was grounded in the transtheoretical model of behavior change [34,35], self-determination theory [36], motivational interviewing [37], and the CBT model of gambling [38]. The transtheoretical model informs on the process of change, self-determination theory and motivational interviewing on the motivations for change, and CBT on concrete strategies for change. Taken together, the intervention worked to elicit and strengthen intrinsic motivations, which are associated with an increased readiness for change and subsequent success [39]. Specific, practical, evidence-based strategies were offered to achieve self-determined goals. These strategies generally fell into the categories of stimulus control (eg, limiting access to gambling opportunities), behavioral activation (eg, replacing gambling with other activities), and cognitive-motivational (eg, reframing cognitive distortions, especially around risk and probability). Goal selection was also based on a harm reduction approach in that users could choose to maintain their current level of gambling, cut down in various ways, or quit completely. Evidence suggests that a pretreatment abstinence goal generally does not confer an advantage over moderation in terms of gambling treatment outcomes, although abstinence may be more popular among those with severe gambling problems [40].

#### Content Development

App content was primarily adapted from paperback and online versions of an original self-directed workbook, *Becoming a Winner: Defeating Problem Gambling* [41]. That workbook was grounded in the same theoretical framework as the current mobile app. Both the paperback and online versions have demonstrated effectiveness for treating gambling problems [29,30,42-46]. Additional educational content was added based on historical user requests for more audiovisual material, as well as evidence for newly developed *Lower-Risk Gambling Guidelines* [47]. The content of the final intervention prototype consisted of a daily check-in survey, 10 interactive activities, and 10 noninteractive educational resources.

#### App Features and User Flow

**Figure 1.**
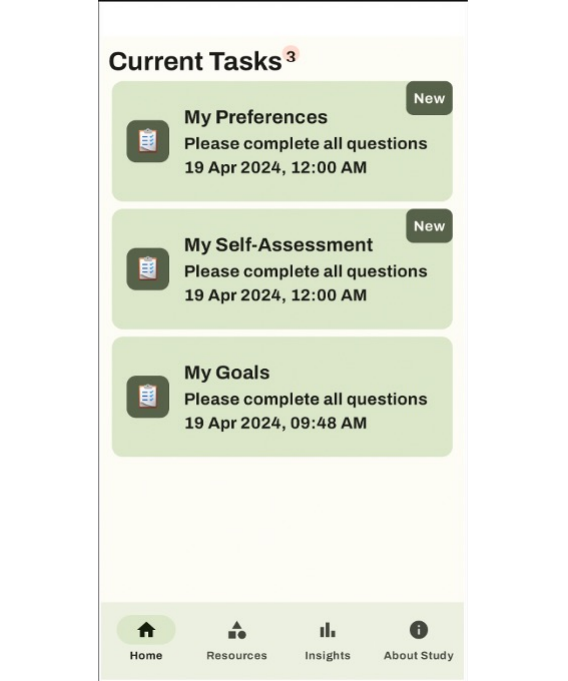
Intervention home page on the first day.

**Figure 2.**
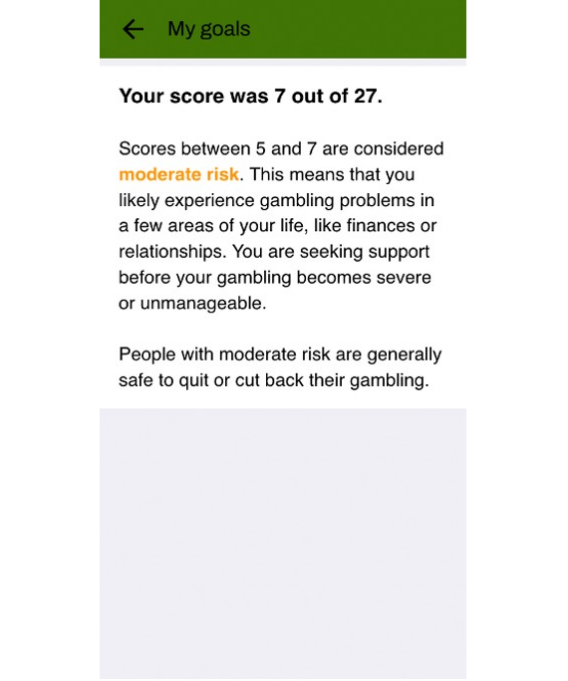
Sample self-assessment feedback.

Immediately upon enrollment, users completed 3 brief mandatory assessments in the app—notification time preference, gambling self-assessment, and treatment goal selection. The display of those assessments is presented in Figure 1. Time preference referred to push notifications for the daily check-ins. The self-assessment contained the 9 PGSI items and specific feedback based on the calculated level of risk (Figure 2), which was intended to inform users when selecting a treatment goal. Goal options included abstinence, moderation, and maintenance. Those who chose moderation or maintenance were given several options for setting limits, such as limiting the number of gambling types or the amount of time or money spent over a specified time frame. These assessments could not be revisited, but users could reach out via email to the research team if they wished to have their responses changed.

Check-ins were prompted once daily via push notifications at the user-selected time. Each check-in began with a reminder of the user’s treatment goal and queried whether the goal was met that day. Users then indicated how many minutes and dollars they gambled. Those who did not gamble were asked to rate their overall mood and gambling urges on 10-point scales based on the past 24 hours. Those who did gamble responded to analogous items that queried mood and urges just before gambling. Those who gambled were also asked to indicate relevant settings (eg, casino and online), types (eg, sports betting and slot machines), and motivations (eg, social and emotional) for their recent gambling. Personal data regarding gambling duration and expenditures were graphed in a separate tab within the app. In total, daily check-ins contained 4 questions for nongamblers and 10 for gamblers.

**Figure 3.**
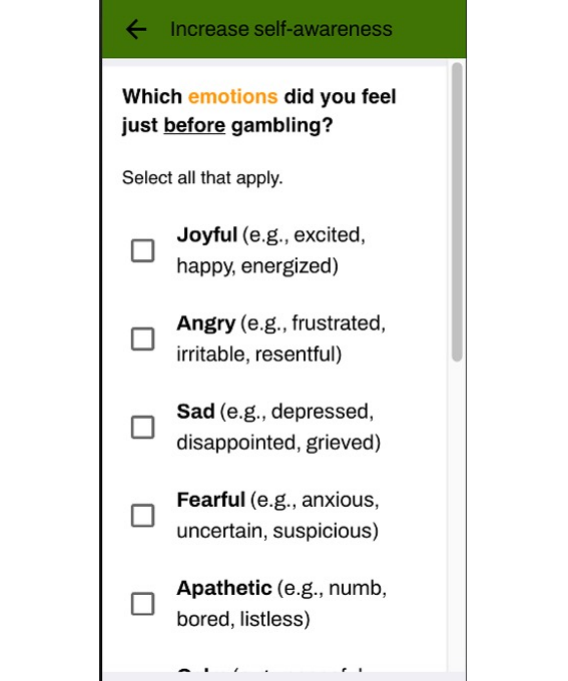
Sample of the multiple-choice response options on the thought record.

Over the first week, interactive intervention activities were triggered based on user responses to check-ins. These recommended activity prompts were delivered 5 minutes after the daily diary was completed. All activities, including untriggered ones, then became available in buffet style after the first week and could be completed as many times as desired and in any order. Each activity included multiple-choice and open-ended text responses. Refer to Figures3  4 for sample responses in the thought record. Responses were then piped into a progress report on the final day. Furthermore, 5 noninteractive educational resources were also available immediately upon enrollment for the duration of the study. The remaining resources became available in the same manner as the interactive intervention activities (ie, based on diary responses in the first week, then available in buffet style in the second week). All app content is presented in Table 1.

**Table 1.** App content and delivery.

App component	Format	Delivery	Intention
Daily diary	Survey	Daily push notifications; on-demand view of expenditure graph	Permit routine self-monitoring; collect data for personalized graphs and activity recommendations
Notification preferences	Survey	Push notification upon enrollment	Assess preference for timing of daily diary push notification
Self-assessment	Survey	Push notification after completing notification preferences	Use responses to provide personalized feedback on gambling problem severity
Treatment goal selection	Survey	Push notification after completing self-assessment	Provide autonomy in goal selection
Thought record	Interactive activity	Push notification first time daily goal is not met	Identify cognitive and emotional antecedents to gambling
Gambling calendar	Interactive activity	Push notification first time daily duration exceeds 60 min	Calculate time and money spent gambling
Decisional balance exercise	Interactive activity	Push notification first day of gambling	Identify costs and benefits of gambling and not gambling
Values card sort	Interactive activity	Push notification first time goal is partially met	Clarify personal values to work towards
Motivation rulers	Interactive activity	Push notification third time goal is not met	Gage confidence, importance, and commitment to change, and identify what would make each stronger
Trigger identification and management	Interactive activity	Push notification first time a trigger is identified	Identify all triggers and reflect on how to manage them
Reflection on past behavior changes	Interactive activity	Push notification when no other activity is triggered	Identify transferable skills from past changes
Making amends with others	Interactive activity	Push notification first time gambling with others	Identify interpersonal harms from gambling and how to ameliorate them
Relapse prevention plan	Interactive activity	Push notification third time goal is met	Plan for future urges and triggers
Progress report	Personalized feedback	Push notification on last day of enrollment	View a summary of all text responses to activities
Gambling and the brain	YouTube video	Immediately available upon enrollment	Understand the neuroscience behind gambling
LRGGs^a^	Read-only web page	Immediately available upon enrollment	Familiarize with responsible gambling limits
LRGGs types of gambling	Read-only web page	Immediately available upon enrollment	Understand different types of gambling and the risk conferred
LRGGs gambling-related harm	Read-only web page	Immediately available upon enrollment	Understand types of gambling-related harm
Common cognitive distortions	Read-only article	Push notification one day after enrollment	Learn about gambling-related cognitive distortions (eg, gambler’s fallacy)
Addictive features of gambling	Read-only article	Push notification three days after enrollment	Learn about addictive features of gambling games and venues (eg, losses disguised as wins)
Cognitive and behavioral coping skills	Read-only article	Push notification five days after enrollment	Refer to a list of coping skills used by past gamblers that helped them reach their goals
Urge surfing	Read-only article	Push notification first time urges rated 6/10 or higher	Learn how to manage urges mindfully
Strategies for limiting access to money and gambling	Read-only article	Push notification first day gambling in multiple settings	Identify strategies for limiting access (eg, voluntary self-exclusion)
List of resources and helplines in Canada	Read-only article	Immediately available upon enrollment	Connect with other resources when self-help is insufficient
App feedback	Survey	Push notification on last day of enrollment	Provide feedback on various app contents

a LRGG: Lower-Risk Gambling Guideline.

**Figure 4.**
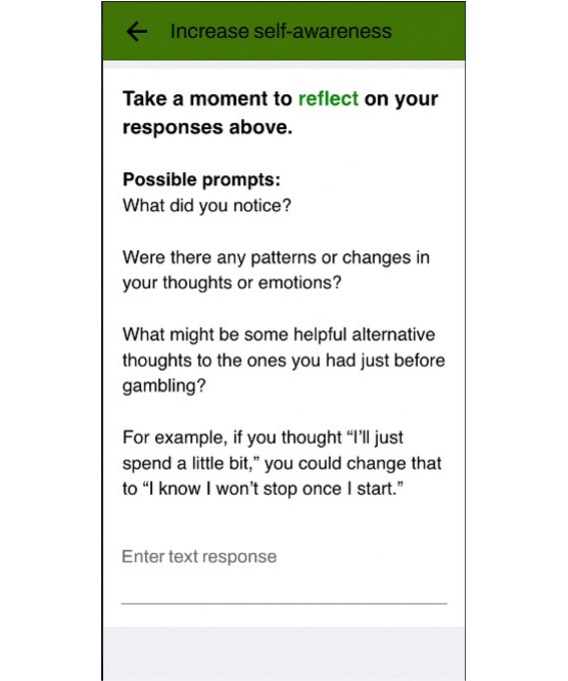
Sample of the open-ended response options on the thought record.

On any given day except the final day, users received a maximum of 2 notifications. The first notification prompted the daily check-in, and the second one prompted 1 relevant activity or resource. If the push notification criteria for multiple activities or resources were met, only the first unseen activity or resource was prompted in order to cap the number of notifications per day. After 1 week, any remaining unseen activities or read-only resources became available. On the final day, after the progress report was viewed, users were prompted to rate each of the activities, resources, and other app features they engaged with throughout the study. They rated each activity and resource on a 4-point Likert scale from “not at all helpful” to “extremely helpful.” Users were enabled to skip any rating if they did not complete the activity or view the resource.

### Measures

#### User Feedback

On the postintervention Qualtrics feedback survey, participants completed the user version of the Mobile App Rating Scale (uMARS), which assesses user perceptions of the intervention’s engagement, functionality, aesthetics, information quality, and subjective quality [48]. Mean subscale scores of 3 or greater are typically considered acceptable for recommending use with a target behavior [49]. Additional questions on the uMARS pertain to overall ratings, user likelihood of paying for the app and recommending it to others, projected use over the next year, and perceived impact on various aspects of behavior change (eg, increased knowledge of problem behavior or importance of behavior change). All 29 quantitative items on the uMARS are ranked on a 5-point Likert scale. The uMARS has demonstrated excellent interrater reliability and internal consistency (Cronbach α=0.90) [48]. We additionally asked users on the Qualtrics survey to rank order the most and least important features of the app.

#### App Usage Data

App usage data were automatically measured within the *MetricWire* platform. These data recorded which diaries and activities were completed and how many times, the duration to complete each activity and diary, and the delay between push notifications and app use. User device type (ie, Apple or Android) was also recorded.

### Data Analyses

No generative AI tools were used in any portion of this manuscript, including the generation, analysis, or presentation of language, content, data, or references. Quantitative data were analyzed using R and RStudio [50]. Results include means and SDs for continuous data as well as counts and percentages for categorical data. There were no missing data on the PGSI or uMARS. App usage data were incomplete by design, as not all content was necessarily used by all participants, and some usage (eg, viewing read-only resources) could not be captured by the platform. Accordingly, analyses of app engagement were conducted using available-case denominators specific to each feature, and no imputation was performed.

Given the formative nature of this study, feasibility and acceptability were primarily evaluated with the uMARS. Consistent with previous use of the uMARS in early-stage app evaluations [49], mean subscale scores of 3 or greater out of 5 were treated as an acceptability heuristic rather than a hypothesis test. Engagement and adherence metrics were not prespecified as stop-go feasibility thresholds; instead, these metrics were summarized descriptively to inform targets for subsequent iterations. The primary metrics of interest were diary completion rate, activity completion rate, and median activity duration.

Qualitative survey data were subjected to a simple content analysis. Responses were reviewed line-by-line, and codes were inductively generated. Codes were then grouped into categories of feedback based on various app features, and minority views were retained. Coding was done by author BWB in consultation with the research assistants who cofacilitated the focus group. Survey data were analyzed separately from the focus group because we did not have ethical approval to link specific survey responses to focus group participants. The intention of these analyses was to contextualize or provide brief examples of quantitative data rather than to add significant new information.

## Results

### Participants

The sample (n=30) had a mean age of 44.0 (SD 12.4) years. Participants were mainly from Ontario (n=10, 33%) or Alberta (n=7, 23%), but all 10 Canadian provinces except Prince Edward Island were represented by at least 1 participant. The majority were White (n=22, 73%), employed (n=19, 63%), female (n=18, 60%), and in a married or common-law relationship (n=16, 53%).

In terms of gambling behavior, 26 (87%) participants regularly gambled in public settings and 12 (40%) in private settings. Additionally, 24 (80%) participants regularly gambled online. They engaged in about 4.6 (SD 2.5) types of gambling, the most popular of which were instant tickets (n=23, 77% participants), lottery tickets (n=21, 70%), video lottery terminals or slot machines (n=19, 63%), bingo (n=18, 60%), and raffles (n=16, 53%). Most participants (n=22, 73%) had ongoing (vs historical) gambling problems. The mean PGSI score of the full sample was 12.7 (SD 6.1), and there was no difference in terms of problem status (*P*=.55). Most participants (n=24, 80%) had previously received some form of support for gambling problems outside the context of a research study, but all had participated in the self-guided web intervention trial from which they were recruited. Only 5 (17%) participants reported they had used a mobile app similar to the current one, and 7 others heard of similar apps but never used them.

### Feasibility

#### App Usage and Compliance

The majority of users (19/30, 63%) accessed the app from an iOS device; the rest used Android. The mean completion rate for daily diaries across users was 51% over 2 weeks (median 64%, IQR 0%‐100%). Across days, the number of diary respondents ranged from 14/30 (47%) on day 9 to 21/30 (70%) on day 5. The mean submission time was 4 PM. Participants submitted 238 of 420 possible diaries (56.7%; odds ratio [OR] 1.3, 95% CI 51.9%‐61.3%). All diary submissions except 3 were started within 5 seconds of the respective push notification. Mean duration for the diary was 14 (SD 8) seconds for users who did not gamble on the diary day and 61 (SD 30) seconds for users who did gamble. In terms of in-app assessments, all users except 1 completed their notification preferences, PGSI self-assessment, and treatment goal selection. Furthermore, 2 users changed the time of their daily diary notification after their initial selection.

Median completion time for intervention activities ranged from 48 (IQR 35-90) to 137 (IQR 93-328) seconds. Means were higher (2-12, SD 5-25 min) because a small subset of users took over 1 hour to complete activities and typically provided thorough open-ended responses. The most frequently completed activity was the reflection on past behavioral changes, which was completed by 25/30 users (83%; OR 5.0, 95% CI 66%‐93%). The next most frequently completed activities were the decisional balance exercise, identification of triggers, making amends, and a relapse prevention plan, all completed by 23/30 users (77%; OR 3.3, 95% CI 59%‐88%). The remaining activities were the thought record (22/30 users, 73%; OR 2.7, 95% CI 56%‐86%), progress report (22/30, 73%; OR 2.7, 95% CI 56%‐86%), gambling calendar (21/30; 70%, OR 2.3, 95% CI 52%‐83%), values card sort (20/30; 67%; OR 2.0, 95% CI 49%‐81%), and importance, confidence, and commitment rulers (17/30; 57%; OR 1.3, 95% CI 39%‐73%). Many participants repeated at least 1 activity, and all activities were repeated by at least 1 participant. The most commonly repeated intervention was the thought record, which 7 of 30 users (23%; OR 0.3, 95% CI 10%-42%) completed 2 to 7 times. Of the 331 total interventions completed, 36% (n=119) were diary-prompted based on specific user data and a subsequent push notification; the rest were user-prompted (on demand). For diary-prompted interventions, the mean delay from notification to activity start time was 82 minutes 29 seconds (SD 248 min 43 s; median 2 min 31 s, IQR 29 s to 32 min 23 s).

#### Technical Difficulties

Some technical difficulties were encountered, but none appeared to prompt disengagement from the app, according to user feedback. The most common issue was improper variable displays (eg, push notifications stated “Hello participantFirstName” instead of actual names). There were also limitations of certain question formats and notification timing options in the *MetricWire* platform. All identified technical difficulties were resolved as soon as possible during the study.

### Acceptability

Feedback was gathered from participants in 3 ways—general feedback on Qualtrics (n=30, 100% response rate), specific feature ratings within the app (n=15, 50% response rate), and a 1-hour virtual focus group. Regarding the focus group, 10 participants were randomly selected and invited to participate. Of those 10, 8 consented to the focus group, 6 ultimately attended, and 2 provided written feedback via email instead. Furthermore, 2 participants did not respond to the invitation.

### General Survey Feedback

Quantitative feedback on Qualtrics was primarily collected via the uMARS (Table 2). Of the 4 objective domains, functionality was rated the highest on a 5-point scale, followed by information, aesthetics, and engagement. The highest-rated subdomains were information credibility, ease of use, and gesture functionality. The lowest-rated subdomains were customization, interactivity, and entertainment value. All subdomains except customization surpassed the acceptability heuristic threshold of 3.0.

**Table 2.** uMARS^a^ ratings out of 5 for each domain and subdomain.

Domain and subdomain	Mean (SD)
Engagement	
Entertainment	3.1 (1.2)
Interest	3.5 (1.1)
Customization	2.8 (0.8)
Interactivity	3.1 (1.0)
Target audience	4.2 (0.5)
Total	3.4 (0.7)
Functionality	
Performance	4.2 (0.8)
Ease of use	4.3 (0.7)
Navigation	4.2 (0.8)
Gestures	4.3 (0.7)
Total	4.2 (0.6)
Aesthetics	
Layout	3.8 (0.9)
Graphics	3.8 (1.1)
Appeal	3.6 (0.7)
Total	3.7 (0.8)
Information	
Quality	4.1 (0.7)
Quantity	4.0 (0.9)
Visual	4.1 (0.7)
Credibility	4.3 (0.8)
Total	4.1 (0.6)

a uMARS: user version of the Mobile App Rating Scale.

Overall, the mean star rating for the app was 3.6 (SD 0.8) stars out of 5. Out of 30 users, 7 (23%) indicated they would be somewhat likely to pay for the app. The remainder were either ambivalent (9/30, 30%), somewhat unlikely (5/30, 17%), or extremely unlikely (9/30, 30%); none were extremely likely. In terms of projected use over the next year, if access were available, 3% (1/30) said never, 17% (5/30) said 1 to 2 times, 40% (12/30) said 3 to 10 times, 33% (10/30) said 10 to 50 times, and 7% (2/30) said more than 50 times. The dispersion of responses was similar when users were asked if they would recommend the app to others with gambling problems; 3% (1/30) said they would not recommend it to anyone, 13% (4/30) said they would recommend it to very few people, 43% (13/30) said some people, 10% (3/30) said many people, and 30% (9/30) said they would definitely recommend it to anyone with gambling problems.

As part of the uMARS, participants were then asked to rate the perceived impact the app would have in various domains. Those who endorsed a clear or strong positive impact (vs somewhat, minimal, or no positive impact) were 63% (19/30) for knowledge about gambling problems, 43% (14/30) for further help-seeking, 40% (12/30) for attitude toward change, 40% (12/30) for motivation to change, 33% (10/30) for importance of change, and 30% (9/30) for concrete change in gambling behaviors. Across all domains, 1 person consistently reported no anticipated positive impact, but the remainder endorsed minimal or somewhat positive impacts. The user with uniformly negative ratings did not provide any qualitative responses to elaborate on their ratings.

In addition to the uMARS, participants were asked to select up to 5 of the most and least important aspects of the app for achieving the primary goal of reducing gambling problems (Table 3). The response options were derived from the uMARS. The aspects of greatest importance were ease of use, interactivity, and quality of information. The aspects of least importance were entertainment value, invitation of user feedback, and overall layout. Aspects of greater disagreement included entertainment value, visual appeal, customization, and amount of information, as these were all rated among the most and least important by at least one-third of users. Similar response variability was observed in the open-ended feedback, which was offered by 23 of 30 (77%) respondents (Table 4).

**Table 3.** Perceived most and least important aspects of the app.

Aspect	Most important (n=30 responses), n (%)	Least important (n=27 responses), n (%)
Entertainment value	11 (37)	16 (59)
Visual appeal	11 (37)	10 (37)
Customization	9 (30)	9 (33)
Interactivity	16 (53)	5 (19)
Invitation of user feedback	8 (27)	12 (44)
Appropriate for target audience	11 (37)	5 (19)
Performance and speed	5 (17)	5 (19)
Ease of use	17 (57)	0 (0)
Overall layout	6 (20)	12 (44)
Amount of information	13 (43)	9 (33)
Quality of information	14 (47)	2 (7)
Credibility of information	13 (43)	4 (15)

**Table 4.** Summary of qualitative content analysis (n=23).

Category	Positive feedback	Critical feedback
Daily diaries or check-ins	Valued for accountability, self-reflection, and self-monitoring (n=3)	Repetitive, effortful, boring, or activating (n=5)
Personalized graphs of time and expenditures	Helpful for self-awareness and motivation (n=2)	—^a^
Triggered recommendations and notifications	—	Some disliked timing or felt interrupted (n=2)
Interactive activities	Thought-provoking (n=1)	Burdensome or insufficient (n=4)
Information and psychoeducation	Credible and useful (n=5)	Mixed views on quantity and format (n=2)
Aesthetics and layout	Clean, simple, and attractive (n=4)	Too minimal or not gamified enough (n=5)
Duration	—	Some wished for more time with the intervention (n=3)
Support structure	—	Some wished for more guidance or support (n=2)
Functionality	Ease of use and good functionality (n=3)	Technical difficulties (n=5)
Perceived impact	Helpful for increasing insight, self-reflection, and motivation (n=7)	Concern that the app added nothing unique beyond what can be found online (n=2)

a Not applicable.

### Specific In-App Feedback

**Figure 5.**
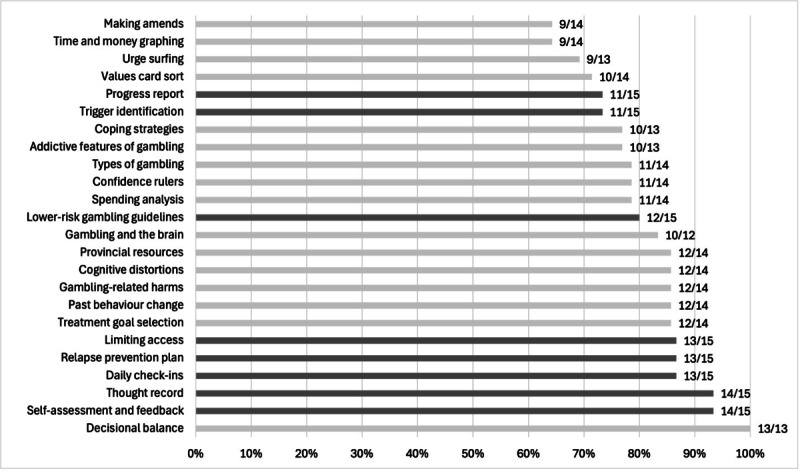
Proportion who rated each activity as somewhat or very helpful (numerators in the figure represent the number of people who rated each activity as somewhat or very helpful; denominators represent the total number of people who rated each activity; those who skipped the question, or those who responded they did not recall completing the activity, were excluded from denominators; and dark gray bars represent activities completed and rated by all 15 feedback respondents).

Feedback was elicited from 15 of 30 participants (50%) within the app on the final day of enrollment. Ratings are summarized below (Figure 5) in terms of the percentage of users who identified each activity as somewhat or very helpful (vs minimally or not at all helpful). All interventions and resources were completed by at least 12 of the 15 users who provided in-app feedback (80%).

### Focus Group Feedback

The sample was too small to evaluate whether focus group attendees and nonattendees differed in their app ratings, so we could not determine whether focus group responses were biased (for or against) the app. However, attendees provided both positive and constructive feedback that was largely consistent with open-ended survey responses.

By far, the most salient feedback from the focus group was the need to expand opportunities for user choice. This conclusion was largely driven by the group’s high variability in individual preferences, which mirrored the variability observed in the open-ended survey responses as well as the lower customization ratings on the uMARS. For instance, some strongly preferred thought-provoking and open-ended reflections, while others gravitated toward more efficient checkboxes or multiple choice. The same pattern was observed with respect to information medium, such that some preferred audiovisual resources, whereas others preferred text. Similarly, some liked the consistency of check-in questions to reduce cognitive load, while others felt disengaged by the repetition. Many users found the check-ins to be the most helpful part of the app, particularly to track spending and remain focused on treatment goals, but 2 strongly preferred other activities and resources. Some users felt overwhelmed by the amount of information requested or provided by the app, while others desired more data tracking or informative resources. One user provided suggestions for modifications to existing resources (eg, display all the same information in both text and visual format). Moreover, 2 users would have liked more time with the app to track their gambling behaviors, and 1 suggested gamification to enhance engagement. Participants provided some feasible technical suggestions for implementing the choice (eg, constantly available rather than time-limited expenditure log).

Areas of greater agreement included the frequency of notifications, utility of tracking and graphing expenditures, and rating scales for mood and urges. Most users found the frequency of notifications (ie, once or twice daily) to be just right. A number of minor technical suggestions were also provided to further enhance ease of use (eg, including an orientation page for first-time users and editing how buttons link to various pages within the app).

## Discussion

The results of this study describe early acceptability and feasibility findings that will inform subsequent modifications and testing on a larger scale. On the whole, users responded positively to the app prototype, including efforts ultimately designed to improve engagement, such as customized notification times, graphs of personal diary data, and tailored recommendations. Users believed the app was credible, easy to use, and appropriate for the target audience – factors which they indicated were among the most important for app developers to consider. The highest-rated activities were the decisional balance exercise, self-assessment feedback, and thought record; the latter was also the most repeated. Median completion time was consistently below 2.5 minutes (IQR 93-328 s) for activities and 1 minute (IQR 38-74 s) for diaries, minimizing the cognitive demand on users. When summed across all activities, activity completion time was similar to the overall 27 minutes spent on a static online intervention with nearly identical content [29]. However, summing the mean activity completion time here still likely underestimates app use, given that it excludes time spent browsing the app or viewing read-only resources. Despite relatively lower ratings on the uMARS regarding entertainment value and aesthetics, the majority of users indicated these factors were among the least important in the effectiveness of the app to help people reduce gambling-related problems. All this feedback is consistent with our own past work [29] and others’ [51]. Overall, the app prototype was rated 3.6 out of 5, and 25 out of 30 users would recommend the app to at least some others struggling with gambling problems. Importantly, however, it cannot be assumed that feasibility or acceptability at this formative stage will translate to engagement or effectiveness at a larger scale or with a different sample composition [51].

The largest gap between suggested importance and actual app delivery was for tailoring factors (eg, interactivity and customization). Indeed, the most consistent theme in the feedback was the lack of consistency, underscoring the need for a more personalized experience. This finding is concordant with other gambling intervention acceptability studies [52,53]. One major area of diverging opinion was related to the information medium and quantity. This critique applies to both the amount of information provided and the amount requested from users through check-ins and activities. Specifically, some users wanted more information and were content to input their data, while others felt overwhelmed by what was already provided and burdened by what was requested from them. Despite these critiques, users agreed on the high quality and credibility of information and overall rated the quantity of information 4.0 out of 5 on the uMARS. This finding is especially positive given that users felt the app could be most impactful in terms of increasing knowledge about gambling problems.

A more pressing area of disagreement related to the perceived utility and willingness to complete the daily check-ins. Many users believed the check-in, along with the associated graphing of personal gambling data and recommended activities and resources, were the most helpful parts of the app. Others found the daily diaries to be tedious and preferred to access activities and resources at their leisure. In fact, two-thirds of app engagement was on demand rather than based on tailored recommendations. In one sense, this finding is promising in that users will access an intervention without requiring prompting. In another sense, it presents a challenge, given that one of the primary means to personalize an intervention is by recommending various activities based on data provided in check-ins. Put another way, more data provided by users yields more tailored recommendations. This problem becomes more salient when considering that the diary completion rate was only 51% over 2 weeks. Diary completion rates have varied substantially in studies using momentary assessments of gambling behavior [17,54-57]. It is likely that with advancements in AI and machine learning (ML), it will be much more feasible to tailor interventions based on dynamically evolving individual needs in real-time, minimizing the cumbersome trial-and-error approach currently used [58]. However, AI and ML often require a very large amount of data to operate. The burden on users to provide data for tailoring may be reduced in part by passive sensor data [59]. Still, AI and ML do not provide a short-term solution, as there remain unresolved concerns about the risks of using AI and ML to inform gambling treatment [60,61] and general mental health treatment [62,63].

In addition to the mixed feedback, several relatively uncontroversial suggestions for improvement were provided by users. These suggestions included displaying more information in both text and visual formats, making the read-only resources more interactive, offering more than 2 weeks with the app for ongoing tracking of gambling data, providing additional information for special populations (eg, Indigenous gamblers) or concurrent substance use, and implementing gamification to enhance engagement. On the last point, it has been cautioned that gamification should strengthen, and not detract from, treatment content [64]. Other areas for improvement to consider include integrating the Lower-Risk Gambling Guidelines throughout the app and querying concurrent substance use and positive coping strategies in the daily check-ins. Based on observed engagement in this formative study, subsequent iterations will prespecify engagement targets (eg, diary completion and activity completion) and evaluate whether refinements improve these metrics.

This study presents some limitations. First, our sample was small and drawn from a highly selective pool of participants who had already participated in previous studies we conducted. These individuals were likely more research-experienced and more motivated relative to first-time intervention users or app store visitors. Given their participation in a similar previous intervention, their feedback was likely more forgiving than feedback from a naïve sample would have been, and thus may not generalize to other subpopulations of gamblers. Moreover, we did not solicit feedback from clinicians, other gambling researchers, or people with at-risk or low-severity gambling problems. Taken together, the feedback we received from our sample may not be representative of all stakeholders or broader population needs. However, as noted, the feedback was relatively consistent with past work. Also, the selection of focus group participants via random number generator, use of a semistructured interview guide, and inclusion of both recurring themes and outlier opinions were implemented to support balanced collection and reporting of feedback.

A second limitation is that this study did not measure the app’s objective influence on engagement or gambling behavior change. While users’ initial perceptions of potential impact are informative, subsequent work will need to measure these variables directly. Third, the qualitative feedback we gathered was relatively succinct and could have benefited from a more thorough mixed methods approach, such as a more thorough interview guide and additional content analyses of transcribed interviews. Fourth, there were limitations inherent to the use of a third-party software to host our intervention. Certain planned features and functionality, particularly those related to personalization, were not feasible within *MetricWire*’s platform. It was also not possible to gather data on the number of people who viewed read-only resources. Some minor technical difficulties were encountered as expected, but *MetricWire* support was responsive, and all difficulties were quickly resolved. While *MetricWire* provided an appropriate and cost-effective means to conduct this initial stage of testing, it would be sensible to develop independent software in the future. Finally, the results of this study do not yet inform on the primary purpose of the app – that is, to enhance engagement and reduce gambling problems.

Overall, this preliminary study provides important data to inform app modifications and testing on a larger scale. In fact, there are 2 ongoing trials using a refined version of this app, both of which will gather feedback and evaluate longer-term engagement and effectiveness in samples that are naïve to the intervention. Participants in this study responded very positively to the app prototype and agreed on its credibility, ease of use, and potential impact, although their familiarity with the content may have made their feedback particularly forgiving. Some areas of disparate opinion were encountered, the most pressing of which pertained to daily diaries, personalization, and aesthetic appeal. This app has the potential to narrow the gap in evidence-based self-guided treatments for gambling problems and provide the help they want and need on demand.

## Supplementary material

10.2196/83430 Multimedia Appendix 1 Interview schedule.

## Abbreviations

AI: artificial intelligence
CBT: cognitive behavioral therapy
ML: machine learning
OR: odds ratio
PGSI: Problem Gambling Severity Index
uMARS: user version of the Mobile App Rating Scale
