# Supplementary material for: Help on Demand, a Self-Directed Mobile App Intervention for Gambling Problems: Development and Usability Study
Source: JMIR Form Res. 2026 Mar 24;10:e83430. doi: 10.2196/83430 (PMC13012611; doi:10.2196/83430)
Supplement: Multimedia Appendix 1 [file formative-v10-e83430-s001.docx]

**Interview Guide**

Interview should be limited to 30 minutes and scheduled around participant availability. Interview should be open-ended and flow according to participant priorities, but the following guideline should be followed. Steps 1, 2, and 7 must be covered in full; steps 3 through 6 can be focused on with greater or lesser emphasis depending on what matters to participants.

1. Introductions. Ask if they have any general questions about their participation. Remind that they consented to being recorded. Begin recording with their expressed consent. If they decline to be recorded at this point, do not record, and ask if they consent to taking notes throughout the interview instead.
2. Provide brief summary/reminder of research project and aims as outlined in the consent form.
3. Ask in an open-ended manner about their general experience with the app. Focus on likelihood of use, strengths and concerns about everyday use, ideas for maximizing user engagement, usefulness of information, ease of use, quality of examples for behavioural skill implementation, technical difficulties.
4. Probe about their responses to the feedback survey, particularly those responses that stand out from others’ (e.g., especially high or low ratings). Ask them to elaborate on their lowest and highest ratings. Example questions:
   1. Are the resources/activities/diaries on the app helpful? Would you want more of them? Less of them?
   2. Had you heard of the LRGGs before this?
   3. How to make the app more engaging/gamified? What do you think about that- negative or positive to take advantage of gambling?
   4. How does it compare to other app use? How generally active on phone are you?
   5. What was the most frustrating technical issue for you?
   6. What did you think of the compliance and insight reports?
   7. What do you think about the time period? How long before bored?
5. Ask about their suggestions for improvement in any of the areas covered during the interview and/or in the feedback survey.
6. Ask in an open-ended manner if they have any further information, suggestions, questions, or concerns they would like to provide.
7. Thank them for their participation and provide information on how and when they will be compensated with their gift card.
